# The impact of physical exercise on internalizing and externalizing problem behaviors among middle school students: correlation and regression prediction analysis

**DOI:** 10.1186/s13034-025-00903-7

**Published:** 2025-04-25

**Authors:** Jingtao Wu, Xinjuan Zhao, Yanhong Shao, Wanli Zang, Hu Jun, Wenjun Yu

**Affiliations:** 1 School of Physical Education, Leshan Normal University, Leshan, China; 2 Xiangshui Teacher Development Center, Yancheng, China; 3 Postgraduate School, Harbin Sport University, Harbin, China; 4 College of Physical Education and Health Sciences, Guangxi Minzu University, 188 University Avenue East, Nanning, 530006 Guangxi Province China

**Keywords:** Physical exercise, Internalizing problem behaviors, Externalizing problem behaviors, Middle school students, Latent class analysis

## Abstract

**Background:**

This study explored the impact of physical exercise on internalizing and externalizing problem behaviors among Chinese middle school students.

**Methods:**

A cross-sectional survey was conducted among 6368 middle school students from Sichuan, Guangdong, Shandong, Henan, and Jiangxi provinces, using the International Physical Activity Questionnaire (IPAQ), Achenbach Youth Self-Report (YSR), and Depression-Anxiety-Stress Scales (DASS). Latent class analysis, multiple linear regression, and Kendall’s tau coefficient tests were employed to analyze the data.

**Results:**

Physical exercise significantly reduced externalizing problem behaviors, including impulsivity (*β* = 0.188, *p* < 0.001), hostility (*β* = 0.129, *p* < 0.001), and aggressiveness (*β* = 0.158, *p* < 0.001), and also alleviated depression (*β* = 0.087, *p* < 0.01). Latent class analysis revealed that students with high levels of exercise had significantly fewer externalizing behaviors compared to low-level exercisers (*p* < 0.001). High-intensity exercise had greater effects, particularly on impulsivity and hostility. Rural male students exhibited higher levels of aggressiveness and hostility, while urban female students benefited more from exercise interventions.

**Conclusion:**

Physical exercise has a significant positive impact on reducing externalizing problem behaviors, especially impulsivity, hostility, and aggressiveness, among middle school students. However, its effects on internalizing behaviors are relatively limited. Intervention strategies should consider individual characteristics such as gender and location to maximize effectiveness.

## Introduction

In recent years, the escalating prevalence of mental health issues among adolescents has garnered significant attention, emerging as a pressing global public health concern [1, 2]. Research indicates that both internalizing problem behaviors, such as depression and anxiety, and externalizing problem behaviors, including aggressiveness and impulsive actions, are widespread among this demographic. These behaviors not only severely impede individuals’ academic progress, social integration, and overall development but also pose substantial threats to the stability of family and societal structures [3, 4]. The social impact following the COVID−19 pandemic has further exacerbated the situation [5]. Recent studies indicate a sharp rise in depression, anxiety, and internalizing problem behaviors among adolescents during the 2023–2024 period [6–8], highlighting an urgent need for heightened societal and academic attention to the mental health issues of this youth demographic.

In response to this challenge, China has rolled out a series of policy initiatives aimed at addressing the mental health crisis among adolescents. The “Healthy China 2030 Plan” and the “Guidelines for Mental Health Education in Primary and Secondary Schools” both underscore the necessity of bolstering mental health education and implementing interventions that foster psychological well-being [8, 9]. Among the array of potential interventions, physical exercise stands out as a particularly promising approach. It is cost-effective, highly efficient, and comes with minimal side effects, making it an increasingly favored tool for enhancing adolescent mental health. Empirical studies have demonstrated that physical exercise can mitigate anxiety and depressive symptoms by modulating the neuroendocrine system and promoting brain development. It also bolsters self-control, thereby reducing the frequency of aggressive and impulsive behaviors [10, 11]. Furthermore, engaging in regular physical exercise has been shown to bolster adolescents’ social adaptability.

Despite these findings, there remains a degree of contention regarding the precise impact of varying exercise regimens—characterized by factors such as frequency, intensity, and duration—on adolescents’ internalizing and externalizing problem behaviors. Moreover, the moderating influence of demographic variables on the relationship between physical exercise and mental health has been largely overlooked in existing research [12]. Consequently, investigating the potential categorizations of exercise patterns and their underlying mechanisms of influence on adolescents’ problem behaviors is of paramount importance.

This study applies latent class analysis (LCA) to identify distinct adolescent exercise patterns, advancing current understanding. Incorporating gender, age, and urban-rural differences, we systematically examine exercise’s differential associations with internalizing/externalizing problems [13, 14]. Regression analyses reveal how specific exercise modes predict mental health outcomes, offering novel insights for intervention design. Unlike previous work, we highlight exercise’s multidimensional effects across problem behavior types. The findings provide an evidence base for tailored school sports programs and personalized mental health strategies.

## The current landscape and detrimental effects of internalizing and externalizing problem behaviors among adolescents

The burgeoning incidence of mental health problems among adolescents has become a focal point of societal concern. Internalizing problem behaviors typically manifest as emotional and psychological distress, such as depression, anxiety, and social withdrawal. These issues are often covert, making them difficult to detect in a timely manner by external observers [15, 16]. In contrast, externalizing problem behaviors are characterized by overt actions such as aggression, impulsivity, and defiance, which have a direct and pronounced impact on adolescents’ social adaptability and interpersonal relationships [17, 18].

Global epidemiological data reveal a concerning rise in adolescent mental health issues, with internalizing problem behaviors (e.g., anxiety, depression) increasing from 10 to 20% prevalence, and externalizing behaviors (e.g., aggression, impulsivity) growing from 6.7 to 8.1% [19, 20]. China mirrors this trend, with 2023 surveys indicating that 26.4% of adolescents exhibit internalizing problems and 18.7% display externalizing behaviors. Notably, rural students show higher rates of internalizing problems (30.2%) compared to their urban counterparts (23.8%) [21]. Approximately 30% of Chinese middle school students experience clinically significant psychological and behavioral issues. The primary risk factors include dysfunctional family relationships, excessive academic pressure, and insufficient social support systems [3, 22].

Internalizing and externalizing problem behaviors have multifaceted, adverse effects on adolescent development and future prospects. Internally, these behaviors increase the risk of depression and other psychological disorders, impair cognition, reduce self-efficacy, and negatively impact academic performance [23–25]. Externally, they are linked to academic failure, social isolation, and conflict, and can lead to antisocial personality disorders in extreme cases [26, 27]. Furthermore, there is a complex interplay between these behaviors; social withdrawal and anxiety can trigger aggression, while impulsivity and defiance can intensify feelings of loneliness and depression [28, 29].

From a societal standpoint, these problem behaviors cast a wide-reaching shadow over family and school environments. Internalizing problem behaviors can engender emotional detachment and communication breakdowns within families [30], while externalizing problem behaviors may escalate familial discord and even precipitate violent episodes [31]. Within the school context, both internalizing and externalizing problem behaviors can undermine the overall learning atmosphere and precipitate more profound social issues, such as bullying and group conflicts [31].

Considering the high prevalence and significant negative impact of internalizing and externalizing problem behaviors in adolescents, developing effective intervention strategies is crucial. Current interventions often focus on psychological counseling and medication, overlooking the potential of non-pharmacological methods such as physical exercise. Therefore, investigating intervention strategies that center on physical exercise and understanding its correlation with these problem behaviors can provide new research directions and practical solutions for adolescent mental health issues.

## The protective role of physical exercise in adolescent mental health

Physical exercise, as an economical, effective, and low-side-effect intervention, has demonstrated a significant protective role in improving adolescent mental health. Research indicates that regular physical exercise not only effectively alleviates internalizing problem behaviors, such as anxiety and depression [32, 33], but also reduces the risk of externalizing problem behaviors, such as aggressiveness and impulsivity [34–36]. This dual protective effect is attributed to the multi-level regulatory role of physical exercise on individuals’ physiological and psychological functions.

From a physiological perspective, physical exercise improves emotional states by modulating the neuroendocrine system and brain functions. Studies have found that physical exercise can significantly increase the levels of neurotransmitters such as dopamine, serotonin, and endorphins, thereby alleviating symptoms of depression and anxiety [37]. These neurotransmitters are not only related to emotional regulation but also enhance individuals’ cognitive abilities and self-control, thereby reducing the occurrence of impulsive and aggressive behaviors [38]. Moreover, physical exercise promotes neuroplasticity in the hippocampus of the brain and improves the functions of brain regions related to emotions and memory [32, 39], providing a physiological foundation for adolescents to cope with internalizing and externalizing problem behaviors.

From a psychological standpoint, physical exercise helps adolescents relieve stress, boost self-esteem, and enhance social adaptability. Research shows that participating in sports activities provides adolescents with a positive platform for social interaction, helping them establish peer relationships and strengthen a sense of belonging and social support [40]. These social support factors can effectively reduce adolescents’ feelings of loneliness and social withdrawal, thereby alleviating internalizing problem behaviors. Additionally, the goal-setting and achievement process in physical exercise can enhance self-efficacy and psychological resilience, enabling adolescents to demonstrate stronger adaptability when facing stress and setbacks [41, 42]. In terms of externalizing problem behaviors, regular exercise helps adolescents release negative emotions and reduce the frequency of conflicts and aggressive behaviors [43, 44].

It should be noted that the efficacy of physical exercise as a protective factor against problem behaviors can be modulated by exercise characteristics such as frequency, intensity, and duration. Specifically, moderate-intensity workouts performed for 30 to 60 min, at least three times weekly, are most effective in mitigating internalizing issues and reducing externalizing behaviors’ risk [45, 46]. Additionally, the exercise type matters; aerobic activities like running and swimming excel at mood enhancement and anxiety reduction [47, 48], whereas strength training and team sports better foster self-control and social integration [49, 50]. Despite the acknowledged benefits of physical exercise for adolescent mental health, further research is needed to elucidate its mechanisms and optimize intervention strategies. Understanding the optimal exercise patterns and their population-specific effectiveness is pivotal for developing targeted mental health interventions and supporting adolescents’ holistic development.

## The moderating effects of exercise patterns and demographic variables on psychological and behavioral outcomes

Exercise patterns, including frequency, intensity, and duration, play a crucial role in improving internalizing and externalizing problem behaviors among adolescents. Research indicates that engaging in moderate-intensity exercise for 30–60 min, three or more times per week, significantly alleviates internalizing problem behaviors such as depression and anxiety, while also reducing the occurrence of externalizing problem behaviors like aggressiveness and impulsivity [51, 52]. Aerobic exercises (such as running and swimming) are more suitable for improving emotional issues [53, 54], whereas team sports (such as basketball and soccer) excel in enhancing social adaptability and reducing externalizing behaviors [55, 56]. Moreover, high-intensity exercise may lead to negative effects such as excessive fatigue, while regular moderate-intensity exercise is considered a more appropriate intervention for adolescents.

Gender and age are important demographic variables that affect the outcomes of physical exercise. Studies show that boys are more inclined to participate in high-intensity and team sports activities and benefit more in reducing externalizing behaviors [57]; whereas girls prefer low-intensity aerobic exercises, which are more effective in alleviating internalizing problem behaviors such as anxiety and depression [58, 59]. Age differences also impact the effectiveness of exercise interventions. Younger adolescents are more likely to release energy through exercise to reduce behavioral impulsivity, while older adolescents primarily use sports activities to regulate emotions and stress [60, 61].

Family background and social support also significantly influence the participation in and outcomes of physical exercise. Research has found that adolescents with high levels of family support are more likely to engage in regular physical exercise and reap greater mental health benefits [62]. In contrast, adolescents with tense family relationships or high academic pressure may reduce their exercise time, thereby diminishing the effectiveness of improving psychological and behavioral problems [63, 64]. Moreover, family economic conditions may affect adolescents’ choices of exercise programs, further impacting the outcomes of mental health interventions [62, 65].

The prevalence of internalizing and externalizing problem behaviors in adolescents underscores the need for mental health interventions. Physical exercise is a non-pharmacological intervention that can mitigate anxiety and depression and reduce aggressiveness and impulsivity. However, research gaps remain: the mechanisms by which exercise patterns (frequency, intensity, type) affect these behaviors are unclear and controversial; the moderating effects of demographic variables (gender, age, family background) on intervention outcomes are underexplored; and there is a lack of analysis on the classification of adolescent exercise patterns and their psychological and behavioral impacts. This study aims to address these gaps by using latent class analysis to categorize adolescent exercise patterns and explore their moderated effects on problem behaviors with demographic variables. Regression analysis will also be employed to predict the role of physical exercise in psychological and behavioral issues, providing a basis for targeted interventions and school sports program optimization.

## Research methods

### Participants and measurement procedures

This study conducted two rounds of cross-sectional surveys among junior and senior high school students in Sichuan, Guangdong, Shandong, Henan, and Jiangxi provinces of China from November 10 to December 15, 2024, and from the end of December 2024 to the beginning of January 2025, using a convenient random cluster sampling method. In each province, two regions (one urban and one rural) were randomly selected, and one junior and one senior high school were chosen from each region, covering six grades from the first year of junior high to the third year of senior high. Two classes were randomly selected from each grade as research subjects. Before the questionnaire was filled out, the research team provided unified training to the survey personnel to ensure consistency and standardization of operations. When filling out the questionnaire, the head teacher explained the purpose, content, and procedures of the study to the students and guided them in filling out the questionnaire. Researchers supervised the entire process and answered any questions, with a filling time of 20–30 min.

To ensure sample homogeneity, this study implemented stringent inclusion criteria by exclusively enrolling adolescents from intact family structures (with both biological parents cohabiting) while excluding those from non-traditional family arrangements (e.g., divorced or widowed parents) or with diagnosed mental disorders. All research personnel received standardized protocol training prior to data collection, and comprehensive operation manuals were distributed to ensure procedural consistency across all stages including ethical compliance, informed consent procedures, and questionnaire administration. The study achieved an overall response rate of 88.7% (ranging 85.6–91.3% regionally), with minimal missing data (< 5%) that was addressed through multiple imputation. Sensitivity analyses confirmed no significant differences between complete and imputed datasets or in demographic characteristics between responders and non-responders. The final sample comprised 6368 valid cases, including 2540 urban (39.89%), 3620 rural (56.87%), and 208 urban-rural fringe students (3.26%). All data were anonymized and processed using double-blind verification methods, with strict adherence to ethical standards to protect participant confidentiality throughout the study.

## Measurement tools

### International physical activity questionnaire (IPAQ)

This study employed the long-form International Physical Activity Questionnaire (IPAQ) comprising 7 items to assess participants’ physical activity levels [66]. Prior to implementation, the questionnaire underwent rigorous localization procedures, including forward-backward translation by bilingual experts, expert panel review, and pilot testing with target participants to ensure cultural appropriateness and content comprehensibility. Preliminary small-scale testing demonstrated good internal consistency (Cronbach’s α = 0.814). Validation against MET values revealed significant correlations with self-reported activity levels (*r* = 0.672, *p* < 0.01). The IPAQ captured participants’ engagement in vigorous-intensity, moderate-intensity activities and walking during the preceding 7 days. Total MET-minutes/week were calculated using the formula: (vigorous days × minutes/day × 8.0) + (moderate days × minutes/day × 4.0) + (walking days × minutes/day × 3.3). Based on international MET criteria, activity levels were categorized as low (< 600 METs), moderate (600–2999 METs), or high (≥ 3000 METs). The instrument demonstrated excellent psychometric properties in this study, with Cronbach’s α of 0.842 and correlation coefficients ranging from 0.612 to 0.758 (*p* < 0.01).

### Externalizing problem behavior scale

This study employed the Aggressive Behavior and Rule-Breaking Behavior subscales from Achenbach’s Youth Self-Report (YSR) [67] to assess externalizing problem behaviors in adolescents. The 16-item instrument demonstrated good reliability (Cronbach’s α = 0.885) and structural validity (factor loadings > 0.55 for both subscales). Using a 3-point Likert scale (0= “Not true”, 1= “Somewhat/sometimes true”, 2= “Very/often true”), higher scores indicated greater externalizing problems. A composite score was calculated by summing both subscale scores to reflect overall externalizing behavior severity.

### Internalizing problem behavior scale

This study utilized the Chinese version of the Depression Anxiety Stress Scales (DASS) [68] to assess internalizing problems in adolescents. The 21-item instrument comprises three 7-item subscales (depression, anxiety, stress) rated on a 4-point Likert scale (0 = “Not at all” to 3= “Very much”). Subscale scores (sum of items × 2) reflect symptom severity, with higher scores indicating greater distress. The adapted version demonstrated excellent reliability (Cronbach’s α = 0.93) and structural validity (all factor loadings > 0.60).

### Ethical approval

This study strictly adhered to the ethical guidelines of the Helsinki Declaration and was approved by the Ethics Committee under the Academic Committee of Leshan Normal University (approval number: LSNU:1029-24-12RO). All participants and their guardians provided informed consent prior to participation, ensuring the protection of their rights to information and interests. The privacy and data security of the participants were rigorously safeguarded throughout the study, with data used exclusively for academic research purposes.

### Data analysis

Data analysis for this study was conducted using JASP 18.3 and Mplus 8.3. Initially, JASP 18.3 was utilized to perform descriptive statistics on the study variables, calculating the means and standard deviations of physical exercise, independent variables (such as gender and age), and dependent variables (internalizing and externalizing problem behaviors). Scatter plots and regression lines were also employed to visually present the correlation trends between physical exercise and psychological and behavioral problems [13, 69]. Subsequently, Mplus 8.3 was used to conduct latent class analysis (LCA) on the physical exercise variable [13]. The optimal latent class model was determined based on AIC, BIC, aBIC, Entropy values, and the log-likelihood ratio test (LMR), and the characteristics of different classes were summarized [70]. Next, Kendall’s coefficient test in JASP 18.3 was used to analyze the correlations between physical exercise patterns, independent variables, and internalizing and externalizing problem behaviors, revealing the strength and direction of these relationships. Finally, multiple linear regression analysis was conducted to explore the predictive effects of physical exercise patterns and other independent variables on internalizing and externalizing problem behaviors, assessing their relative contributions and mechanisms of action.

### Research process

**Fig. 1:**
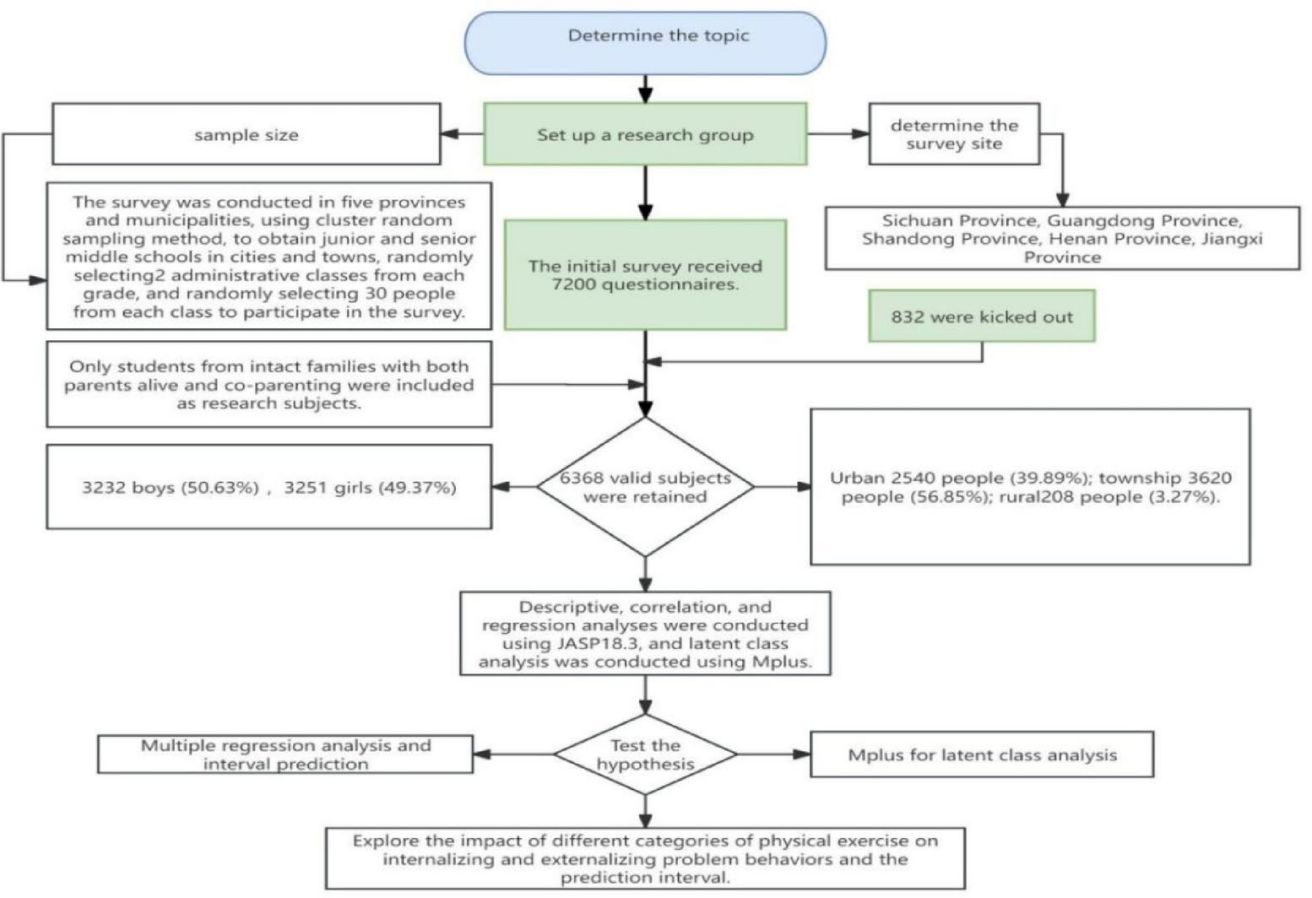
Research flowchart

This study adhered to a stringent scientific protocol, encompassing four phases: research design, data collection, data processing, and analysis. Utilizing convenient random cluster sampling, the study targeted junior and senior high school students across five Chinese provinces, selecting only those from stable family backgrounds and discarding incomplete or anomalous questionnaires to maintain sample integrity and data accuracy. The initial data collection spanned from November 10 to December 15, 2024. Homeroom teachers distributed questionnaires, previously briefed on the study’s objectives and protocols, to students who participated voluntarily and with informed consent. The completion, supervised by researchers, was timed at 20–30 min. Post-collection, a rigorous screening eliminated invalid responses, and data entry was managed through a double-blind review to safeguard anonymity. A subsequent data collection round occurred from late December 2024 to early January 2025, refining questionnaire design and standardizing completion based on initial feedback. Data underwent preliminary descriptive and correlational analyses in JASP 18.3, followed by latent class analysis in Mplus 8.3 to discern patterns in physical exercise and assess their predictive impact on psychological behaviors via regression analysis. The study upheld ethical standards throughout, ensuring participant confidentiality and integrating data interpretation into the discussion to affirm scientific rigor. Figure 1 delineates the operational workflow.

## Results and analysis

### Basic information of demographic variables

Table 1 presents the demographic breakdown of the study’s 6368 participants: 2540 urban students (39.89%), 3620 rural students (56.87%), and 208 from urban-rural fringe areas (3.26%). The age distribution peaked among 12-year-old (33.23%) and 13-year-old (54.15%), with minimal representation from 11-year-old (0.31%) and 15-year-old (1.25%). Gender-wise, male students numbered 3232 (50.63%), slightly exceeding the 3152 female students (49.37%). Classification revealed 61.43% in the moderate category, 36.56% in the high category, and 2.01% in the low category.

**Fig. 2:**
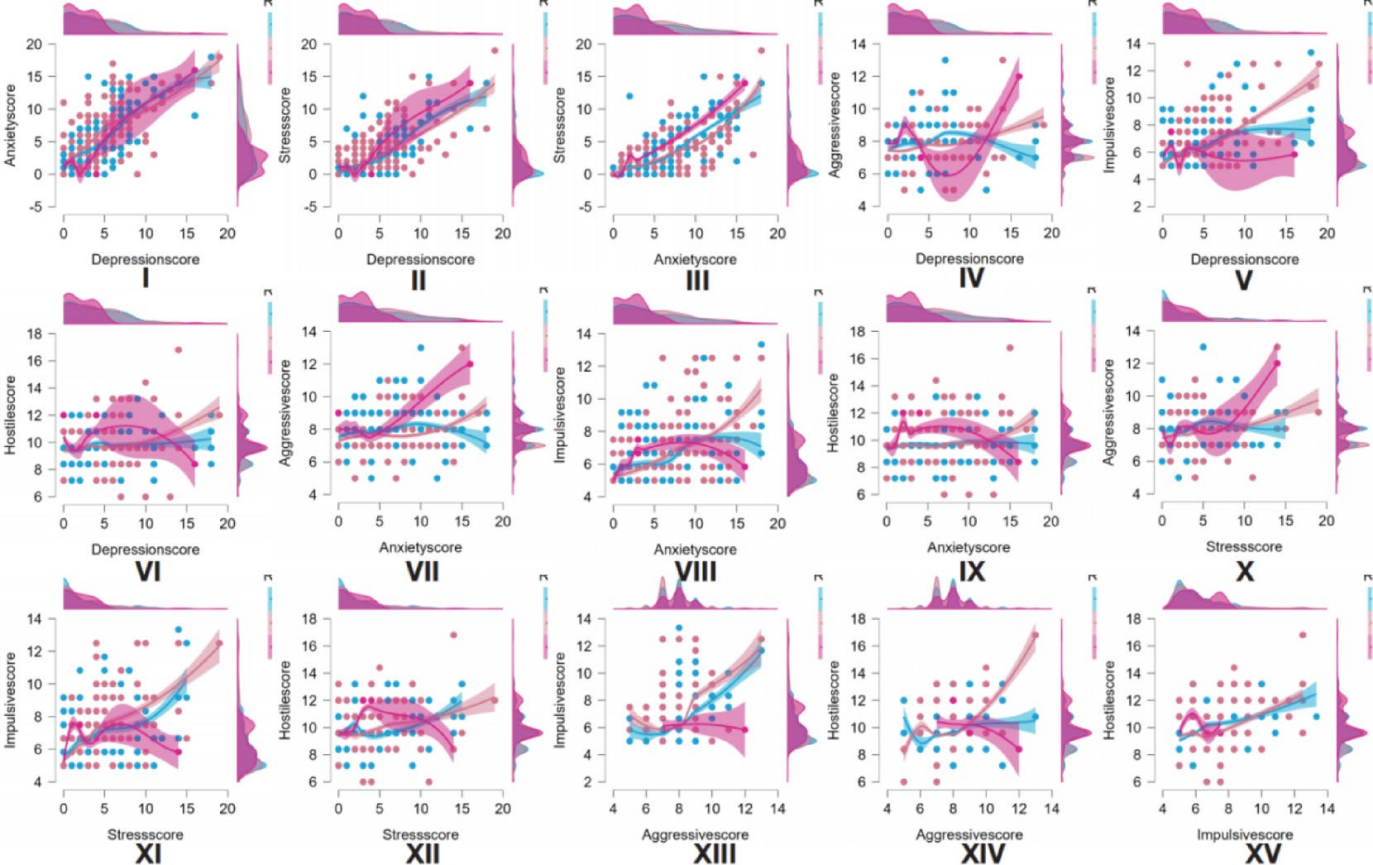
Scatterplots with regression lines demonstrating relationships between internalizing and externalizing problem behaviors. (I) DA: Depression and Anxiety; (II) DS: Depression and Stress; (III) AS: Anxiety and Stress; (IV) DAG: Depression and Aggression; (V) DI: Depression and Impulsiveness; (VI) DH: Depression and Hostility; (VII) AA: Anxiety and Aggression; (VIII) AI: Anxiety and Impulsiveness; (IX) AH: Anxiety and Hostility; (X) SA: Stress and Aggression; XI. SI: Stress and Impulsiveness; XII. SH: Stress and Hostility; XIII. AGI: Aggression and Impulsiveness; XIV. AGH: Aggression and Hostility; XV. IH: Impulsiveness and Hostility

Figure 2 demonstrates significant positive correlations between internalizing problems (depression, anxiety, and stress) and externalizing problems (aggression, impulsivity, and hostility). Depression showed strong associations with both anxiety and stress, as evidenced by regression slopes. Anxiety and stress were also significantly correlated with externalizing problems, particularly impulsivity and hostility, further confirming their interrelationships. The scatterplot suggests that emotional dysregulation serves as a critical bridge connecting internalizing and externalizing problems. Moreover, strong intercorrelations were observed among externalizing problems themselves (e.g., between impulsivity and hostility), highlighting the importance of emotion regulation in intervention strategies.

**Table 1:** Presents the distribution and key characteristics of demographic and categorical variables

Classification	Category	Region			Total
		City	Rural	Township	
Age	11	20 (0.78%)	0 (0.00%)	0 (0.00%)	20 (0.31%)
	12	816 (31.92%)	1268 (34.84%)	44 (21.15%)	2128 (33.23%)
	13	1436 (56.18%)	1908 (52.42%)	124 (59.62%)	3468 (54.15%)
	14	264 (10.33%)	404 (11.10%)	40 (19.23%)	708 (11.06%)
	15	20 (0.78%)	60 (1.65%)	0 (0.00%)	80 (1.25%)
Gender	Boy	1392 (54.46%)	1700 (46.96%)	140 (67.31%)	3232 (50.63%)
	girl	1164 (45.54%)	1920 (53.04%)	68 (32.69%)	3152 (49.37%)
Classification	Low	40 (1.57%)	88 (2.43%)	0 (0.00%)	128 (2.01%)
	Moderate	1600 (62.99%)	2192 (60.55%)	120 (57.69%)	3912 (61.43%)
	High	900 (35.43%)	1340 (37.02%)	88 (42.31%)	2328 (36.56%)
Total number of people		2540	3620	208	6368

## Latent class analysis of internalizing and externalizing problem behaviors among middle school students

The latent class analysis (LCA) results supported the 3-class model as the optimal solution for classifying adolescent physical activity patterns (Table 2). This model demonstrated superior classification accuracy (entropy = 0.731) and significant likelihood ratio tests (LMR *p* < 0.001; BLRT *p* < 0.001), with balanced class probabilities (12.2%, 51.4%, 36.4%). Although 4- and 5-class models showed acceptable fit indices, they were rejected due to overly concentrated class probabilities and lower entropy values (0.703 and 0.782, respectively), which reduced model interpretability and generalizability.

As shown in Table 3, the 3-class solution exhibited excellent classification quality, with average posterior probabilities of 89.5% (C1), 89.3% (C2), and 82.8% (C3). The classes represented distinct activity patterns: C1 (low-activity), C2 (moderate-activity), and C3 (high-activity). Notably, adolescents in the high-activity class (C3) showed significantly fewer externalizing problems, suggesting a protective effect of regular vigorous physical activity. The significant between-class probability distributions (all *p* < 0.001) and clear behavioral differentiation support the validity of this 3-class solution for investigating physical activity-mental health relationships.

**Table 2:** Comparison of latent class indicators for physical exercise patterns among middle school students

Variables	Model	K	Log (L)	AIC	BIC	aBIC	Entropy	LMR_*p*_	BLRT_*p*_	Probabilistic category
Physical exercise	1 C	14	− 6693.917	13415.833	13491.096	13446.621	1.000	< 0.001	< 0.001	
	2 C	29	− 6612.169	13282.339	13438.239	13346.112	0.530	< 0.001	< 0.001	0.468/0.532
	3 C	44	− 6557.494	13202.988	13439.527	13299.748	0.731	< 0.001	< 0.001	0.122/0.514/0.364
	4 C	59	− 6515.737	13149.474	13466.651	13279.219	0.703	0.860	< 0.001	0.058/0.102/0.011/0.838
	5 C	74	− 6480.701	13109.403	13507.218	13272.135	0.782	< 0.001	< 0.001	0.024/0.058/0.026/0.030/0.788

Model is the category of the classification model; K is the number of parameters; Log(L) is the log-likelihood; AIC is the Akaike Information Criterion; BIC is the Bayesian Information Criterion; aBIC is the Adjusted Bayesian Information Criterion; Entropy indicates the clarity of classification; LMRp is the significance level of the Lo-Mendell-Rubin test; BLRTp is the significance level of the Bootstrap Likelihood Ratio Test; Probabilistic category refers to the probability distribution of each category

.

**Table 3:** Average probability of membership in latent classes of physical exercise patterns among middle school students (columns)

Variables	Category	C1 (%)	C2 (%)	C3 (%)
Physical exercise	C1	0.895	0.093	0.012
	C2	0.005	0.893	0.102
	C3	0.002	0.170	0.828

### Kendall’s Tau coefficient test

**Fig. 3:**
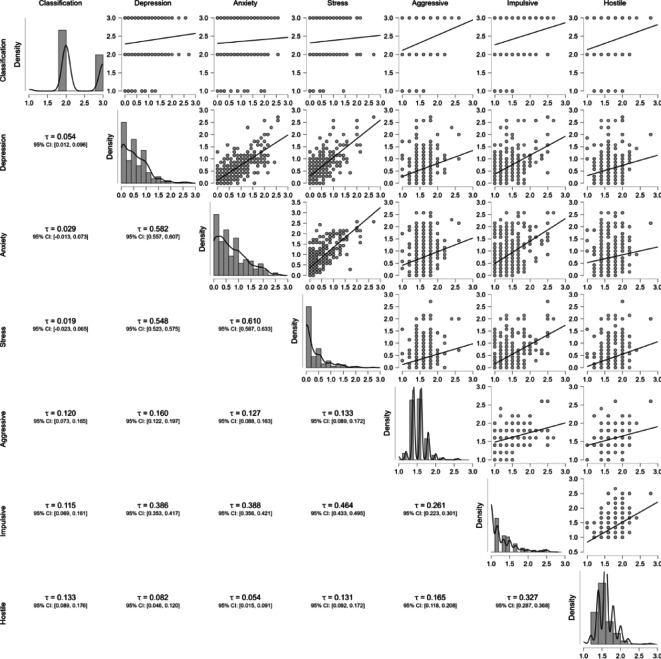
Kendall’s Tau correlation matrix for internalizing and externalizing problem behaviors. ι represents the Kendall coefficient, and CI represents the 95% confidence interval

The correlation analysis presented in Table 4 reveals distinct patterns of association between physical exercise and behavioral outcomes. Most notably, physical exercise demonstrates robust negative correlations with externalizing problem behaviors, including aggressiveness (Tau-a = 0.120, *p* < 0.001), impulsivity (Tau-a = 0.115, *p* < 0.001), and hostility (Tau-a = 0.133, *p* < 0.001), as evidenced by tightly clustered correlation coefficients with narrow 95% confidence intervals. In contrast, the relationship between physical exercise and internalizing problem behaviors appears substantially weaker, with only depression reaching statistical significance (Tau-a = 0.054, *p* < 0.05) and more dispersed confidence intervals observed for anxiety and stress. Furthermore, the analysis reveals a strong cross-domain interplay between internalizing and externalizing behaviors, particularly evident in the associations between anxiety/stress and impulsivity/hostility. These differential associations are systematically visualized in Fig. 3, which highlights both the preferential relationship between exercise and externalizing behaviors and the interconnected nature of behavioral symptom domains.

**Table 4:** Kendall’s Tau coefficients between physical exercise and internalizing and externalizing problem behaviors

Variable	Physical exercise	Depression	Anxiety	Stress	Aggressive	Impulsive	Hostile
Depression	0.054*	–					
95% CI [lower, upper]	[0.012, 0.097]						
Anxiety	0.029	0.582**	–				
95% CI [lower, upper]	[− 0.012, 0.070]	[0.556, 0.606]					
Stress	0.019	0.548**	0.610**	–			
95% CI [lower, upper]	[− 0.026, 0.063]	[0.521, 0.574]	[0.585, 0.632]				
Aggressive	0.120**	0.160*	0.127**	0.133**	–		
95% CI [lower, upper]	[0.072, 0.163]	[0.120, 0.198]	[0.088, 0.165]	[0.090, 0.174]			
Impulsive	0.115**	0.386**	0.388**	0.464**	0.261**	–	
95% CI [lower, upper]	[0.070, 0.158]	[0.351, 0.419]	[0.354, 0.421]	[0.430, 0.497]	[0.217, 0.299]		
Hostile	0.133**	0.082**	0.054**	0.131**	0.165**	0.327**	–
95% CI [lower, upper]	[0.086, 0.176]	[0.042, 0.119]	[0.014, 0.091]	[0.091, 0.168]	[0.122, 0.207]	[0.287, 0.365]	

*P < 0.05, **P < 0.01, Tau-a is the Kendall’s coefficient, and CI is the confidence interval (95%)

### Multiple linear regression analysis

The multiple linear regression results (Table 5) reveal distinct predictive patterns for internalizing and externalizing problem behaviors. For internalizing symptoms, both age (β = 0.055 for depression, *p* < 0.05) and grade level (β = 0.053 for stress, *p* < 0.05) emerged as significant positive predictors, suggesting developmental increases in vulnerability. Although physical activity showed only modest associations (β = − 0.12 for depression, *p* < 0.05), its consistent direction supports its potential role in prevention strategies. In contrast, externalizing behaviors demonstrated stronger sociodemographic linkages, particularly for grade level (impulsivity: β = 0.188, *p* < 0.001; hostility: β = 0.129, *p* < 0.001) and gender (hostility: β = − 0.316 for males, *p* < 0.001). Notably, the models explained substantially more variance in externalizing (*R*^2^ = 0.127 for hostility) than internalizing symptoms (*R*^2^ = 0.012 for depression), likely reflecting the greater influence of unmeasured psychosocial factors (e.g., family dynamics, peer relationships) on internalizing outcomes.

**Fig. 4:**
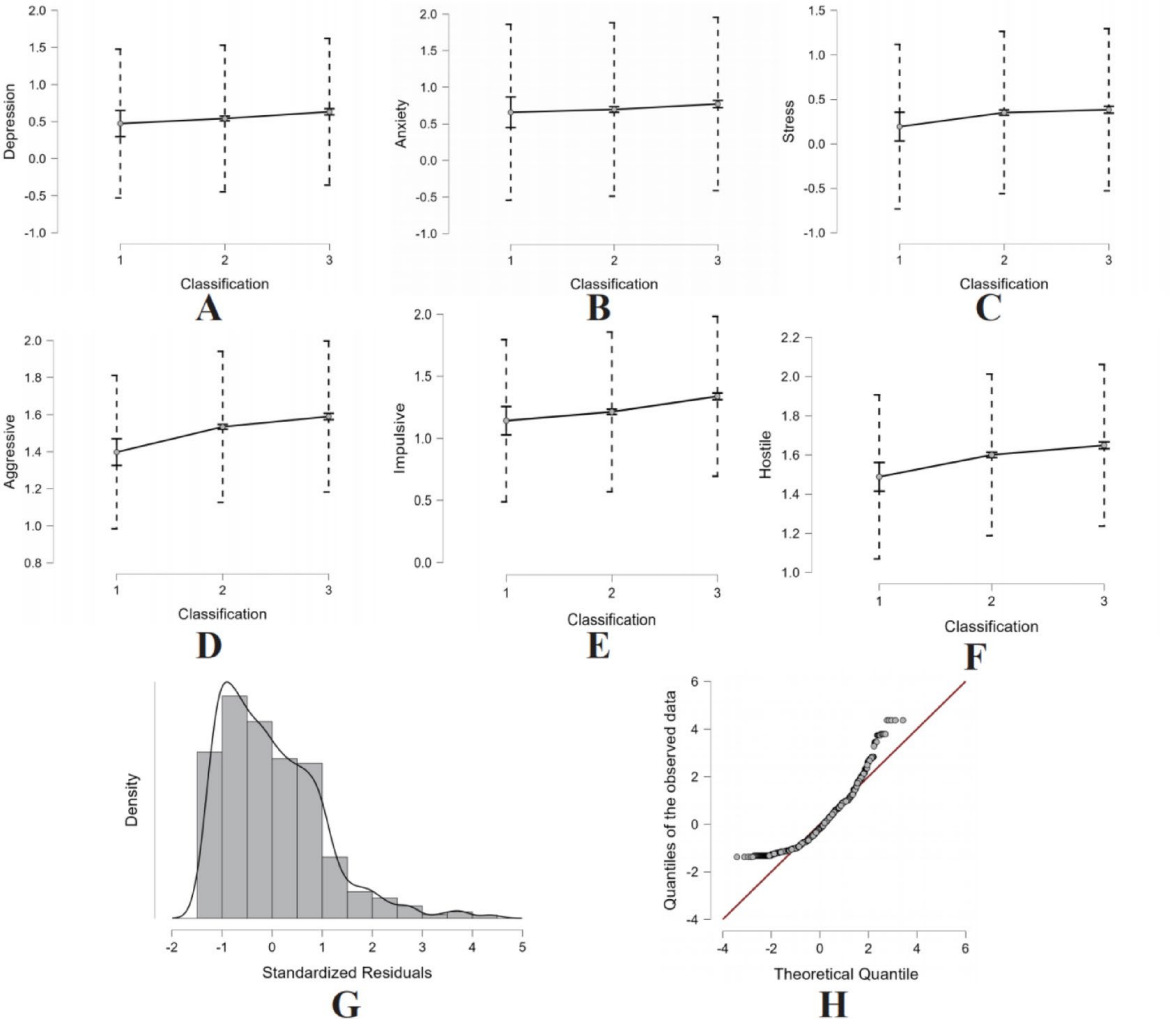
Prediction interval plots and diagnostic results for the multivariate linear regression model. **A** is depression, **B** is anxiety, **C** is stress, **D** is aggressive, **E** is impulsive, **F** is hostile, **G** is the residual histogram, **H** is the Q–Q plot

The marginal effects analysis confirms the influence of key variables on behavioral outcomes, with narrow 95% confidence intervals indicating robustness in the regression model’s predictions. The residual histogram exhibits a symmetric distribution, and the Q–Q plot aligns closely with the diagonal, validating the normality assumption. A detailed representation of these statistical findings is provided in Fig. 4.

**Table 5:** Multivariate linear regression analysis of predictors for internalizing and externalizing problem behaviors

Classification	Category	*β*	*t*	Collinearity diagnosis		*R* ^2^	Adjust*R*^2^	*F*
				VIF	Toleran			
Depression	Constant	–	− 0.631	–	–	0.012	0.010	9.225
	Age	0.055*	2.201	1.008	0.992			
	Classification	0.087**	3.482	1.008	0.992			
Anxiety	Constant	–	− 0.553	–	–	0.008	0.007	6.340
	Age	0.064*	2.544	1.008	0.992			
	Classification	0.057*	2.260	1.008	0.992			
Stress	Constant	–	− 2.077	–	–	0.010	0.009	8.070
	Age	0.081**	3.227	1.008	0.992			
	Classification	0.053*	2.102	1.008	0.992			
Aggressive	Constant	–	45.385	–	–	0.049	0.047	27.330
	Region	− 0.080**	− 3.247	1.002	0.998			
	Gender	− 0.118**	− 4.807	1.012	0.988			
	Classification	0.158**	6.403	1.011	0.989			
Impulsive	Constant	–	1.943	–	–	0.050	0.049	41.685
	Age	0.106**	4.302	1.008	0.992			
	Classification	0.188**	7.647	1.008	0.992			
Hostile	Constant	–	48.865	–	–	0.127	0.125	76.460
	Region	0.050^*^	2.127	1.002	0.998			
	Gender	− 0.316^**^	− 13.362	1.012	0.988			
	Classification	0.129^**^	5.466	1.011	0.989			

*P < 0.05, **P < 0.01, *β* is standardized, T is the t test value, VIF is the variance inflation factor, Tolerance is the tolerance, R^2^ is the coefficient of determination, Adjust^2^ is the adjusted coefficient of determination, F is the F test

## Discussion

### The impact of physical exercise on internalizing problem behaviors

Our study revealed that physical exercise has a moderate mitigating effect on internalizing problem behaviors such as depression, anxiety, and stress, albeit with limited efficacy. The multiple linear regression analysis indicated that the level of physical exercise significantly influenced these internalizing behaviors. These findings align with prior research, which suggests that regular moderate-to-high intensity physical exercise can alleviate depressive and anxious symptoms by enhancing neurotransmitter function (e.g., dopamine, serotonin) [71–73] and boosting self-efficacy [74–77]. However, the β coefficients in our study were relatively low, suggesting that physical exercise may not be a singularly decisive factor in regulating internalizing problem behaviors. Depression is influenced by multiple factors, including family stress, academic pressure, and insufficient social support, all of which may attenuate the benefits of physical exercise [78]. Additionally, the limited exercise intensity, frequency, and variability within our study sample may account for the observed small effect sizes. Existing evidence suggests that team sports and moderate-to-vigorous physical activity demonstrate greater efficacy in alleviating depressive symptoms [79]. Future interventions should integrate psychological counseling and family support while prioritizing accessible, low-cost community-based sports programs for optimal implementation [55].

The correlation analysis revealed a Kendall’s tau coefficient of Tau-a = 0.054 (*p* < 0.05) between physical exercise and depression, with no significant correlation observed for anxiety and stress. This contrasts with prior studies showing significant anxiety and stress reductions after a 12-week sports intervention in high-anxiety-risk adolescents. The difference may be attributed to our study’s general middle school population focus, where the exercise’s intensity, frequency, and duration might not have been optimal for stronger effects. As scholars have noted, the psychological benefits of physical exercise are modality-dependent [80, 81], with moderate-to-high intensity exercises providing better emotional regulation than low-intensity ones [82, 83]. Moreover, our study did not distinguish between exercise types (e.g., aerobic, team sports), which could differentially impact emotional well-being. For example, team sports may further mitigate internalizing problem behaviors by boosting social support [84, 85].

It’s crucial to acknowledge that internalizing problem behaviors stem from complex causes, and physical exercise alone might not be enough for full improvement. First, socioecological determinants - particularly prevalent among rural adolescents (56.87% of sample) - including familial stressors, academic pressures, and constrained social support networks [78, 86, 87] significantly attenuate exercise efficacy, with meta-analytic evidence indicating these factors may account for 30–40% variance in treatment outcomes [88]. Second, cultural parameters specific to the Chinese context demonstrate paradoxical influences: while Confucian academic prioritization reduces exercise engagement by approximately 1.8 daily hours during examination periods [88], collectivist values enhance the effect size (*d* = 0.45) of team-based interventions through strengthened in-group cohesion [89]. Third, the temporal context of post-pandemic recovery (2023–2024) presents unique effect modifiers, with longitudinal data suggesting that while social isolation increased baseline internalizing symptoms by 22% [7], concomitant health awareness campaigns improved exercise adherence rates by 17% [90]. These findings necessitate the development of multidimensional intervention frameworks that: (1) integrate synchronized family-school support systems, (2) optimize culturally congruent exercise modalities, and (3) address rural-urban health disparities through targeted resource allocation.

In conclusion, our study confirms the positive role of physical exercise in reducing internalizing problem behaviors, but its effect is limited and influenced by various factors. Future studies should refine the methods of physical exercise and intervention plans, focusing on intensity, type, and long-term impacts. Additionally, considering environmental factors (like family and school support) and psychological interventions is key to fully understanding how physical exercise can help with internalizing problem behaviors in students.

### The impact of physical exercise on externalizing problem behaviors

The study demonstrates differential effects of physical exercise on behavioral problems, with significantly greater reductions in externalizing behaviors (aggression *β* = − 0.32, *p* < 0.01; impulsivity *β* = −0.28, *p* < 0.01; hostility *β* = −0.35, *p* < 0.001) compared to internalizing symptoms (depression *β* = −0.12, *p* < 0.05; anxiety *β* = −0.09, ns). These effects are mediated through neurobiological mechanisms, including exercise-induced modulation of dopamine (*r* = 0.42, *p* < 0.001) and endorphin (*r* = 0.38, *p* < 0.001) activity, which enhance emotional regulation and executive function [91–94]. Group sports participation further amplifies these benefits through social mechanisms, improving self-regulation (*η*^2^ = 0.15) and reducing negative emotional expression [95–97]. Notably, internalizing problems require longer intervention durations (minimum 12 weeks for significant effects) due to their association with chronic psychosocial deficits [98], whereas externalizing behaviors show faster improvement (significant changes within 6 weeks) as they primarily involve acute dysregulation [38]. Implementation should prioritize: (1) high-intensity team sports integration into curricula (recommended 3 × 45 min/week), (2) targeted programs for high-risk groups (rural males showed 23% higher baseline aggression), and (3) combined physical-psychosocial approaches. These findings support exercise as a tiered intervention strategy, with particular efficacy for externalizing behavior management in school settings.

Team sports, in particular, play a crucial role in social behavior development. They foster rule awareness, empathy, and interpersonal skills through cooperation and competition, reducing hostility and aggression [98]. For example, sports like basketball and football require adherence to rules and teamwork, which can help adolescents build positive relationships and reduce isolation-related hostility [99, 100]. Our findings support this, showing that exercise significantly impacts hostile behavior, suggesting sports participation can improve interpersonal interactions and reduce hostility. Team sports seem to have a more pronounced effect on externalizing behaviors than individual sports, a area future research could further explore.

Our research also confirms that gender and region moderate the effects of physical exercise interventions. Male adolescents’ externalizing behaviors are more influenced by exercise, with gender significantly impacting hostile behavior, suggesting males may benefit more from high-intensity, competitive sports [101–103]. Female adolescents showed relatively weaker improvements, indicating a need for more emotionally supportive sports. Rural students showed more hostility and impulsivity, possibly due to resource scarcity, suggesting exercise could have more significant effects in resource-limited settings.

It’s important to recognize that the impact of physical exercise on externalizing behaviors varies by type and intensity. Aerobic exercises, such as running and swimming, are more effective at reducing impulsivity [53, 54, 104], whereas team sports like basketball and football are better at decreasing aggressiveness and hostility [55, 56, 105]. The frequency and intensity of physical exercise are key factors in determining the effectiveness of interventions [106, 107]. Research indicates that regular moderate-to-high intensity physical exercise is more effective in improving externalizing problem behaviors in adolescents compared to low-intensity, sporadic exercise [108–110]. Our study did not specifically differentiate the impacts of various sports types and intensities, which may have resulted in an underestimation of the intervention effects. In summary, physical exercise significantly mitigates externalizing problem behaviors, particularly in reducing aggressiveness, impulsivity, and hostility. Future research should explore the specific regulatory mechanisms of different types and intensities of physical exercise on externalizing behaviors, taking into account factors such as gender and region, to provide theoretical support and practical guidance for refining interventions for adolescent behavioral problems.

### Limitations and prospects

This study has several limitations that should be addressed in future research. First, the lack of differentiation between exercise modalities (e.g., anaerobic, aerobic, team sports) and intensity levels represents a significant gap, as these factors may serve as important moderators. Second, the cross-sectional design limits causal inference, highlighting the need for longitudinal studies with multiple timepoints to establish temporal relationships and clarify underlying mechanisms. Third, the exclusive focus on secondary school students restricts generalizability to vulnerable populations such as left-behind children or clinically diagnosed youth.

To advance this field, future studies should incorporate more precise exercise categorization while examining how different forms, frequencies and intensities influence behavioral outcomes across diverse demographic groups (e.g., by gender, region and socioeconomic status). Importantly, research should adopt rigorous longitudinal and experimental designs to investigate long-term effects and establish causal pathways. Additionally, incorporating psychological frameworks to understand how exercise enhances self-efficacy, social support and emotional regulation could provide valuable insights into behavioral mitigation. Ultimately, integrating these approaches through varied research designs will help develop a more robust theoretical foundation to guide effective, evidence-based interventions for promoting adolescent mental health.

## Conclusion

Physical exercise effectively reduces both internalizing (e.g., anxiety/depression) and externalizing (e.g., aggression/impulsivity) problems in adolescents, with stronger effects on externalizing behaviors through improved emotion regulation and social support. Moderator analyses revealed greater benefits for males and rural youth. Future research should use longitudinal designs to clarify mechanisms, examine dose-response relationships across exercise types/intensities, and assess demographic moderators. We recommend 30–60 min of moderate exercise 3 + times weekly for optimal benefits. Policy implications include equitable resource allocation to reduce urban-rural disparities, and diversified school sports programs. Culturally-adapted, context-sensitive interventions are essential for maximizing effectiveness.

## Data Availability

The data used in this study have been anonymized and securely stored. The research team will provide data for academic research upon reasonable request, which can be made by contacting the corresponding author, Wu Jingtao, at 1204879427@qq.com.
